# Care Dependency in Patients with Heart Failure: A Cross-Sectional Study in Spain

**DOI:** 10.3390/ijerph17197042

**Published:** 2020-09-26

**Authors:** Raúl Juárez-Vela, Ángela Durante, Begoña Pellicer-García, Antonio Cardoso-Muñoz, José María Criado-Gutiérrez, Isabel Antón-Solanas, Vicente Gea-Caballero

**Affiliations:** 1 Research Group GRUPAC and Research Institute IDI-Paz, School of Nursing, University of La Rioja, 26004 Logroño, Spain; raul.juarez@unirioja.es; 2 Department of Biomedicine and Prevention, University of Rome Tor Vergata, 00133 Rome, Italy; angela.durante@uniroma2.it; 3 Aragón Health Care Service, Primary Care Center of Andorra, 44500 Andorra, Spain; beg2008@hotmail.es; 4 Institute of Neurosciences of Castile and León, Faculty of Nursing and Physiotherapy, University of Salamanca, 37007 Salamanca, Spain; 5 Institute of Neurosciences of Castile and León, Faculty of Medicine, University of Salamanca, 37007 Salamanca, Spain; jmcriado@usal.es; 6 Department of Physiatry and Nursing, Faculty of Health Sciences, University of Zaragoza, 50009 Zaragoza, Spain; 7 Research Group GREIACC, Health Research Institute La Fe, Nursing School La Fe, Adscript Center of University of Valencia, 46026 Valencia, Spain; gea_vic@gva.es

**Keywords:** care, heart failure, dependency, nursing

## Abstract

*Background*: Heart failure (HF) is a progressive and debilitating condition that represents an ever-growing problem for health systems worldwide. HF patients feel that they are a burden on their families, they feel socially isolated and have a low perception of their health. Accordingly, the objectives of this study were to: (1) to explore the profile of care dependency in a representative sample of Spanish HF patients through the Care Dependency Scale (CDS), and (2) to identify correlates of care dependency in this population. *Material and Methods*: We performed a cross-sectional study of 187 patients admitted for HF decompensation to the Hospital Clínico of Zaragoza (Spain). *Results*: Only 15% of our sample was highly or completely dependent on care from others. More specifically, our results indicate that HF patients felt a greater level of dependency on care from others when it comes to moving, getting dressed and undressed, maintaining good personal hygiene, participating in daily and recreational activities and being continent. *Conclusions*: We find association between the CDS categories that present a low score for care dependency in HF patients and the patients’ physical deterioration.

## 1. Introduction

Heart failure (HF) is a progressive and debilitating condition that represents an ever-growing problem for health systems worldwide. It affects more than 5 million people in the USA, and over 10 million people in Europe. Heart Failure prevalence ranges between 0.2% and 0.4% in the general population and increases progressively with age, representing 1%, 10% and 17.4% of patients aged >40, >70 and >85, respectively [1]. Clinical expression of this syndrome includes dyspnoea and fatigue, limits quality of life (QOL), and it is frequently associated with acute exacerbations and recurrent hospital admissions [2,3]. This entails the progressive deterioration of physical function, limiting the patients’ capacity to perform activities of daily living (ADL) such as dressing, grooming/personal hygiene and shopping. As a result, HF patients feel they are a burden on their families, they feel socially isolated and they report a low perception of their health [4]. In recent years, care and protection of the dependent population have become a major challenge for health systems worldwide [5,6]. Although care dependency has frequently been associated with aging [7] patients with advanced HF reported experiencing to have an impaired health status that may result in increased care [8,9]. Furthermore, according to Janssen and Koberich [10,11] care dependency is a determinant of quality of life and survival in patients with HF and, for this reason, regular assessment of HF patients’ symptom burden and care dependency should be undertaken in order to identify patients who are at risk of an impaired health status [9,10]. Research on the prevalence and degree of dependency of Spanish HF patients is scarce. Nurses have a responsibility to identify patients who are at risk of an impaired health status. Accordingly, the objectives of this study were to: (1) explore the profile of care dependency in a representative sample of Spanish HF patients through the Care Dependency Scale (CDS), and (2) identify correlates of care dependency in this population

## 2. Materials and Methods

### 2.1. Sample

A cross-section of 187 patients was admitted for HF decompensation to the Hospital Clínico Lozano Blesa of Zaragoza (Spain), between January 2017 and December 2017. The New York Heart Association (NYHA) functional classification of heart failure is widely used in practice and in clinical studies and was used to determine the severity of functional limitations in our sample. All of our participants were classed as: (1) class II, slight limitation of physical activity, without symptoms at rest, the normal activity causes fatigue, palpitations, or dyspnoea; (2) class III, marked limitation of physical activity, without symptoms at rest; any physical activity causes symptoms; or (3) class IV, symptoms of heart failure are at rest and increase with any physical activity [12].

Inclusion criteria were: (1) being aged 18 years old or older, (2) achieving a score ≥4 in the six-item cognitive impairment test, (3) giving informed consent to participate in the study. The following patients were excluded from the study: (1) patients under the age of 18 years old, (2) patients with cognitive impairment, (3) patients who refused to give informed consent to participate. Ethical approval for the present study was obtained from the Clinical Research Ethics Committee of Aragón (CP13/2015).

### 2.2. Tools

In 1996, Dijkstra, Buist and Dassen [13] developed the CDS with the aim of producing a tool to assess the needs and level of dependency of disabled patients and patients with dementia. The psychometric properties of the CDS have been thoroughly evaluated in previous studies. Additionally, this instrument has been validated on different populations, and it has been translated into more than 16 languages including German, Italian, Norwegian and Spanish [14]. According to Dijkstra, Smith and White’s Manual for the CDS [15], this scale is easy to use and quick to complete, with patients taking an average of 15 min. Specifically, the CDS includes 15 categories, namely (1) eating and drinking, (2) continence, (3) body posture, (4) mobility, (5) day/night pattern, (6) getting dressed and undressed, (7) body temperature, (8) hygiene, (9) avoidance of danger, (10) communications, (11) contact with others, (12) sense of rules and values, (13) daily activities, (14) recreational activities and (15) learning activities, and all categories are marked using a 5-point-scale with responses ranging from 1 (completely dependent) to 5 (almost independent). Achieving a CDS sum score ≤68 indicates that the patient is dependent on care from others, whereas a high sum score means that the patient is almost independent of care.

### 2.3. Statistical Analysis

Sociodemographic variables were described using frequency distributions for qualitative data and mean ± standard deviation (SD) for quantitative data. HF patients’ levels of dependency for each of the categories were described using the median value and interquartile range (IQR) and mean ± typical deviation. In addition, we calculated the distribution of the different dependency categories. Student’s t-test were used to analyze differences for continuous variables, and a Chi-square test or Fisher’s exact test were used to analyze categorical variables. We used binary logistic regression in order to calculate the level of dependency based on those variables which were significant or clinically relevant. The Wald stepwise selection method was used to select the most optimal subset of independent variables. Odds Ratio (OD) was calculated with a confidence interval of 95%.

All calculations were performed using the STATA/SE software version 14.0 (StataCorp LLC, College Station, TX, USA) and *p*-values less than 5% were considered statistically significant.

## 3. Results

A total number of 203 patients agreed to participate in the study. However, 16 participants (7.8%) were rejected due to incomplete data. A final sample of 187 HF patients admitted to the hospital between January and December 2017 took part in the study. The mean age was 81,06 (SD: ± 8.82), and gender distribution was balanced with 94 men (50.30%) and 93 women (49.70%).

The majority of patients (78.40%) were managed at the heart failure unit site within the same hospital, whereas only 21.20% were followed-up by their general practitioners (GPs), as can be observed in Table 1. It is important to highlight the fact that only 56 patients (30.40%) had not reported hospital admissions in the last 12 months; the average length of stay was 11.23 days (TD: ± 9.43).

Results from the CDS are shown in Table 2. Median scores for each of the items show that HF patients’ level of dependency was relatively low (score of 4) in 10 out of the 15 categories; patients reported feeling almost independent in 5 categories, namely body posture, body temperature, communications, sense of “rules and values” and daily activities. Furthermore, 120 patients (64.1%) felt they were almost or completely independent of care from others, with only 28 participants (14.9%) feeling very or completely dependent on their caregivers.

The CDS categories with the lowest mean score (3.72–3.90) were mobility, getting dressed and undressed, hygiene, daily activities, recreational activities and continence Table 3. Similar results were obtained when looking specifically at HF patients who were greatly or completely dependent on others (Table 4), with patients reporting difficulties in items requiring some degree of physical activity (i.e., mobility, day/night pattern, getting dressed and undressed, hygiene and recreational activities). According to the total sum scores on the CDS, 76 (40.60%) patients were independent and 111 (50.4%) patients presented some degree of dependency. According to the total sum scores on the CDS, 76 (40.60%) patients were independent and 111 (50.4%) patients presented some degree of dependency. Table 4 shows the sociodemographic characteristics of the sample, as well as both care dependency groups.

There were some differences between the patients classified as independent and those who reported some degree of care dependency on others. In particular, independent patients were mostly men (60.50%; *p*-value = 0.020), and they were younger (average age 77.46 ± 9.76) than patients who were care dependent (average age 83.05 ± 7.20) (*p*-value ≤ 0.001). In addition, level of education and alcohol consumption (10.50% compared to 1.80%) was higher than for dependent patients; interestingly, their alcohol consumption was also higher. Alcohol consumption is defined as an average weekly consumption greater than 30 g for men and 20 g for women.

The regression model presented in Table 5 shows the profile of patients who, according to the CDS score (>68), were independent of care. As can be observed, they were generally younger with a higher level of education and consumed more alcohol than those who were care dependent.

## 4. Discussion

Few studies demonstrate relations between the Care Dependency Scale and Heart Failure Patients. Our study demonstrates that HF patients reported a low level of care dependency or they were almost independent of care from others. Only 15% of our sample was highly or completely dependent on care from others. Our results are similar to the results reported in previous studies with a similar sample and the same measurement tool [9,11]. More specifically, our results indicate that HF patients feel a greater level of dependency on care from other individuals in terms of moving, getting dressed and undressed, maintaining good personal hygiene, participating in daily and recreational activities and being continent. This finding is in agreement with a previous study of HF patients’ care dependency during their hospital admission [10], and with a study by Incalzi et al. [16] that measured care dependency of HF patients 14 days before hospital admission. As pointed out by Seo et al. [17], the presence of dyspnoea, which is common in HF patients, causes a variable degree of disability. Furthermore, as suggested by these authors, patients with dyspnoea have difficulty dressing and cleaning themselves. An added problem to dyspnoea is urinary incontinence, which is the most frequent comorbidity in patients with HF due mostly to treatment with diuretics and beta-blockers; it significantly impacts their ability and it has a significant impact on their ability to perform their ADL [18]. With regard to gender, higher prevalence values for comorbidities in women have been reported recently in the literature. Temporal changes in epidemiology suggest an increasing comorbidity burden and a higher prevalence in women, leading to more dependence. Women with heart failure are older and more likely than men to have hypertension, kidney failure, obesity, depression, and more severe symptoms, but appear to have better overall survival [19].

Further investigations are necessary to analyze the relationship between urinary incontinence and the CDS categories in HF patients. This study demonstrates that the CDS is a useful instrument in the assessment of care dependency in HF patients. Specifically, the different categories of the CDS provide valuable insight into the needs of these patients, their family and also the environment. In addition, it allows healthcare professionals to perform an in-depth assessment of the patients’ levels of dependency, facilitating the individualization of the care plan for HF patients, especially those individuals with a high level of frailty and dependency. Additionally, our results suggest that there may be a relationship between HF patients’ level of dependency and their ability to undertake physical exercise, evidenced by the fact that most of our subjects expressed a difficulty to undertake ADL, such as getting dressed and undressed, mobility and hygiene. For this reason, we believe that programs that promote the creation and implementation of protocols and interventions aimed at improving HF patients’ health status and disability are important. In particular, we argue that HF patients may benefit from multidisciplinary programs which promote mobility, address depression and adjustment disorder, and train patients and their caregivers in those activities of daily living which require a high level of physical activity.

## 5. Conclusions

There is an association between the CDS categories presenting a low score for care dependency in HF patients and associated with the patients’ physical deterioration, as well as other comorbidities such as dyspnoea and urinary incontinence. Accordingly, healthcare professionals should strive to identify and address these comorbidities in order to improve HF patients’ level of care dependency on others. More studies are necessary to clarify the relationship between these and other comorbidities, and HF patients’ level of dependency and capacity to self-care.

### Limitations

Patients with some degree of cognitive impairment are frequently excluded from these studies [18]. However, not only is cognitive impairment prevalent in this population [10,20,21,22], but, according to Koberich [10], it plays an important role regarding the degree of care dependency, and it is related to disease instability. We excluded from our sample HF patients with cognitive impairment, and recognize that it may have resulted in an underestimation of care dependency in this population.

## Figures and Tables

**Table 1:** Heart failure (HF) management.

		Frequency	Percentage
Place			
	Heart failure unit within hospital	145	78.40
	Primary care	39	21.10
	Other	1	0.50
Hospital admissions in last 12 months			
	0	56	30.40
	1	91	49.50
	2	22	12.00
	3	12	6.50
	4	3	1.60
Length of stay in the last 12 months (M)(SD)		11.23 ± 9.43	

**Table 2:** HF patients’ level of dependency for each of the Care Dependency Scale (CDS) categories.

	Mean	Standard Deviation (SD)	Median	P25	P75
Eating and drinking	3.96	±1.25	4	3	5
Continence	3.90	±1.26	4	3	5
Body posture	4.02	±1.23	5	3	5
Mobility	3.72	±1.29	4	3	5
Day/night pattern	3.97	±1.26	4	3	5
Getting dressed and undressed	3.82	±1.29	4	3	5
Body temperature	4.14	±1.17	5	3	5
Hygiene	3.83	±1.25	4	3	5
Avoidance of damage	3.98	±1.24	4	3	5
Communications	4.12	±1.19	5	4	5
Contact with others	4.14	±1.16	5	4	5
Sense of rules and values	4.10	±1.17	5	3	5
Daily activities	3.87	±1.28	4	3	5
Recreational activities	3.89	±1.30	4	3	5
Learning activities	3.97	±1.21	4	3	5

**Table 3:** Classification in the CDS.

	Frequency	Percentage
Category 1. Patient is completely dependent on care from others (missing all initiative to act; therefore, care and assistance is always necessary)	10	5.3
Category 2. Patient is to a great extent dependent on care from others (many restrictions to act independently, therefore, to a great extent dependent on care and assistance)	18	9.6
Category 3. Patient is partially dependent on care from others (there are restrictions to act independently, therefore, partially dependent on care and assistance)	39	20.9
Category 4. Patient is only to a limited extent dependent on care from others (few restrictions to act independently, therefore, only to a limited extent dependent on care and assistance)	44	23.5
Category 5. Patient is almost independent on care from others (almost everything can be done without assistance)	76	40.6

**Table 4:** Sociodemographic characteristics.

		Total Sample	CDS Dependent ≤68		CDS Independent >68			*p*-Value
		Frequency	Percentage	Frequency	Percentage	Frequency	Percentage	
Gender								0.020
	Male	94	50.30	48	43.20	46	60.50	
	Female	93	49.70	63	56.80	30	39.50	
Age; average (SD)		81.06 ± 8.82	83.05 ± 7.20		77.46 ± 9.76			<0.001
Marital status								0.525
	Single	12	6.40	6	5.40	6	7.90	
	Married	86	46.00	48	53.20	38	50.00	
	Divorced	1	0.50	1	0.90	0	0.00	
	Widower	88	47.10	56	50.50	32	42.10	
Level of education								<0.001
	Primary	164	87.70	107	96.40	57	75.00	
	Secondary	11	5.90	3	2.70	8	10.50	
	Vocational education	1	0.50	0	0.00	1	1.30	
	General Certificate	5	2.70	0	0.00	5	6.60	
	University	6	3.20	1	0.90	5	6.60	
Employment								0.046
	Employed	3	1.50	3	2.70	0	0.00	
	Self-employed	5	2.50	0	0.00	4	5.30	
	Retired	193	95.10	107	96.40	71	93.40	
	Unemployed	2	1.00	1	0.90	1	1.30	
Nationality								0.228
	Spanish	185	98.90	111	100.00	74	97.37	
	Other	2	1.10	0	0.00	2	2.63	
Persons living in the house								0.247
	0	52	27.80	28	25.20	24	31.60	
	1	86	46.00	53	47.70	33	43.40	
	2	30	16.00	20	18.00	10	13.20	
	3	13	7.00	5	4.50	8	10.50	
	4	2	1.10	1	0.90	1	1.30	
	Religious community	4	2.10	4	3.60	0	0.00	
Financial status								0.052
	Has more than enough to live on	38	20.30	16	14.40	22	28.90	
	Has enough to live on	140	74.90	89	80.20	51	67.10	
	Has financial difficulties	9	4.80	6	5.40	3	3.90	
Tobacco								0.337
	No	174	94.10	101	92.70	73	96.10	
	Yes	11	5.90	8	7.30	3	3.90	
Alcohol								0.010
	No	175	93.60	107	98.20	68	89.50	
	Yes	10	5.60	2	1.80	8	10.50	

**Table 5:** Model of logistic regression.

	Odd Ratio	*p*-Value	Confidence Interval 95%
Age	0.92	<0.001	0.89–0.96
Level of education	2.16	0.005	1.26–3.73
Alcohol	6.82	0.020	1.34–34.53
